# Complementary Treatment of the Common Cold and Flu with Medicinal Plants – Results from Two Samples of Pharmacy Customers in Estonia

**DOI:** 10.1371/journal.pone.0058642

**Published:** 2013-03-06

**Authors:** Ain Raal, Daisy Volmer, Renata Sõukand, Sofia Hratkevitš, Raivo Kalle

**Affiliations:** 1 Department of Pharmacy, University of Tartu, Tartu, Estonia; 2 Estonian Literary Museum, Tartu, Estonia; St. Jude Children's Research Hospital, United States of America

## Abstract

The aim of the current survey was to investigate the complementary self-treatment of the common cold and flu with medicinal plants among pharmacy customers in Estonia. A multiple-choice questionnaire listing 10 plants and posing questions on the perceived characteristics of cold and flu, the effectiveness of plants, help-seeking behaviour, self-treatment and sources of information, was distributed to a sample of participants in two medium size pharmacies. The participants were pharmacy customers: 150 in Tallinn (mostly Russian speaking) and 150 in Kuressaare (mostly Estonian speaking). The mean number of plants used by participants was 4.1. Of the respondents, 69% self-treated the common cold and flu and 28% consulted with a general practitioner. In general, medicinal plants were considered effective in the treatment of the above-mentioned illnesses and 56% of the respondents had used exclusively medicinal plants or their combination with OTC medicines and other means of folk medicine for treatment. The use of medicinal plants increased with age and was more frequent among female than male respondents. Among Estonian-speaking customers lime flowers, blackcurrant and camomile were more frequently used, and among Russian speaking customers raspberry and lemon fruits. Regardless of some statistically significant differences in preferred species among different age, education, sex and nationality groups, the general attitude towards medicinal plants for self-treatment of the common cold and flu in Estonia was very favourable.

## Introduction

The common cold is one of the most frequent minor illnesses in the world. Caused by 200 identified types of viruses, the common cold is primarily associated with rhinoviruses [1]. Common cold symptoms may include nasal congestion, an acute cough and/or sore throat [2]. The common cold is distinct from flu, associated with the influenza virus. A fever greater than 38°C and generalized aches and pains are the best predictors of a diagnosis of flu [3]. In the popular approach, both the described illnesses are perceived as similar conditions [4], [5]. The common cold and flu are predominantly self-diagnosed and self-medicated with OTC medicines and/or herbal products [6],[7].

Nowadays complementary medicine, including herbal medicine, is becoming an increasingly popular health care approach, which has been used for both general maintenance of health and for treatment of minor illnesses [8]. In Europe several herbs of different plant species have been used against flu and the common cold: lime and elder flowers, meadowsweet flowers and herb, purple echinacea aerial parts and roots, wild rose, blackcurrant and sea buckthorn fruits, lemon juice, etc [9]. In Russia and Estonia garlic, ramson, onion, raspberry, cranberry, Iceland moss, cowslip primrose aerial parts and roots, plantain species leaves, yarrow, oregano, aerial parts of common and creeping thyme, coltsfoot leaves, willow bark, etc. have also been used for centuries [10],[11].

Estonia is a small country in Northern Europe, one of the three Baltic States, bordering with Finland, Russia and Latvia. About 3/4 of its 1.3 mil population are Estonian speaking, while the remaining population mostly speaks Russian as their mother-tongue. Estonian people have long traditions in the use of medicinal plants and, due to neighbouring Russia, the traditional medicinal plants of both Europe and Russia have been consumed.

Of almost 1500 native and over 700 introduced plant species [12], Estonians utilized over 500 taxa in the period 1888–1994 [5]. Although within the century the proportion of medicinal plants requiring human intervention increased and the use of taxa unfavourable to human impact decreased, overall knowledge on the use of medicinal plants remained high among the Estonian speaking population [13]. Currently in Estonian alternative medicine there are two main means of obtaining information on the use of medicinal plants: traditions or literary sources [14]. Also, some people rely on previous personal experience or information provided by a health care professional, usually a pharmacist [15],[16].

The aim of this study was to evaluate the complementary self-treatment of the common cold and flu with medicinal plants among pharmacy customers in Estonia. We argue that different demographic groups prefer to use different medicinal plants for self-treatment of the common cold and flu.

## Methods

### Survey design

The study was undertaken in two different regions of Estonia – in the capital city of the biggest island of Estonia, Kuressaare, where the majority of permanent residents are Estonian speaking, and in the capital city of Estonia, Tallinn, where the number of Russian speaking permanent residents is higher. In both towns the survey was undertaken in one community pharmacy classified as a medium size pharmacy (approx. 30,000 prescriptions per year) by an independent researcher. From June to August 2011, a sample of pharmacy customers participated in the survey. In both towns the pharmacy customers who had bought prescription or OTC medicine were invited to participate in the survey, the main difference being that in Kuressaare the majority of survey participants were Estonian speaking and in Tallinn, Russian speaking. The research assistant explained the purposes of the study and oral informed consent was received from all interviewed pharmacy customers. In Kuressaare 185, and in Tallinn 174 survey instruments were distributed, with a request to complete the questionnaire at the pharmacy or return it within one week. Due to the lack of time to complete the survey instruments at the pharmacy and to unreturned survey instruments, 150 completed survey instruments were collected from each pharmacy.

### Survey instrument

The survey instrument was developed and discussed by a panel of researchers with a pharmaceutical background and extended knowledge in phytotherapy, ethnobotany and social pharmacy. A copy of the survey instrument can be obtained from the corresponding author.

The survey instrument consisted of multiple-choice items related to (1) perceived characteristics of the common cold and flu, (2) action taken after common cold or flu symptoms have been identified, (3) a list of ten medicinal plants, with a request to provide additional information about the form of use, effectiveness and means of collecting, (4) general perceived effectiveness of medicinal plants against the common cold and flu, (5) an information source about the use of medicinal plants, and (6) demographic characteristics of the respondent.

To compile the list of medicinal plants included in the survey instrument, a literature search was undertaken and a pilot test was run among 13 randomly selected community pharmacists, who selected ten of the more frequently suggested plants against the common cold and flu out of twenty proposed medicinal plants. The content validity and comprehensibility of the survey instrument was pre-tested by ten randomly selected pharmacy customers of participating community pharmacies.

Minor changes to the wording of the items were made, based on feedback received. No samples of medicinal plants were used, but the names of medicinal plants included in the survey were well known and univocal for the speakers of both languages.

The following ten most popular herbs were included in the survey instrument: *Allium sativum* L. (garlic), *Allium cepa* L. (onion), *Zingiber officinale* Rosc. (ginger rhizoma), *Citrus x limon* (lemon), *Echinacea purpurea* (L.) Moench (purple echinacea aerial parts, roots and their preparations), both *Chamomilla recutita* (L.) Rauschert and *C. suaveolens* (Pursh) Rydb. (german chamomile and pineapple weed flowers), both *Tilia cordata* Mill. and *T. platyphyllos* Scop. (lime flowers), *Cetraria islandica* (L.) Ach. (Iceland moss), *Ribes nigrum* L. (blackcurrant fruits, leaves and twigs) and *Rubus idaeus L*. (raspberry fruits and twigs).

### Statistical analysis

Initial data were coded, inserted and stored in an MS Excel database. Most data analysis were performed in SPSS (Statistical Package for Social Sciences, Chicago, IL), while the analysis of the means of used medicinal plants was performed in statistical program R [17]. Frequencies were calculated and cross-tabulation was performed to evaluate the correlation between the use and perception of medicinal plants and demographic characteristics (spoken language, age, gender and education). Linear regression analysis was used; level of statistical significance was set at p≤0.05.

## Results

### Symptoms of and action taken to treat the common cold and flu

Respondents (for details on demographic characteristics see Table 1) perceived the common cold and flu as a combination of several symptoms. The most frequent ones were tiredness, weakness, muscle and joint pain (17%), followed by fever, headache and cough with rhinitis (10%). The rest of the respondents presented other different combinations of described symptoms.

**Table 1:** Socio-demographic characteristics of study population.

	Estonian speaking respondents n = 139*	%	Russian speaking respondents n = 138*	
*Age (years)*				
15–35	37	26.6	38	27.5
36–50	53	38.1	39	28.3
51–65	28	20.1	36	26.1
>65	21	15.1	25	18.1
*Gender*				
Female	103	74.1	97	70.3
Male	36	25.9	41	29.7
*Education*				
Ground school	18	12.9	9	6.5
Secondary school	31	22.3	24	17.4
Secondary school education with specialization	47	33.8	55	39.9
High school	43	30.9	48	34.8
Other	0	0	2	1.4

* 150 completed survey instruments were collected in both Kuressaare and Tallinn. Of those, 9 respondents did not specify the language spoken at home, 11 respondents used both languages and 3 respondents used a language other than Estonian or Russian at home. The mean response rate was 84%, being 81% in Kuressare and 86% in Tallinn.

Female respondents mentioned the whole complex of symptoms more often, and stressed fever with tiredness, while male respondents described fever, headache and cough with rhinitis more frequently (p = 0,004). Both Estonian and Russian speaking respondents presented equally general symptoms of the common cold and flu. However, Estonians listed tiredness as a symptom more frequently, whereas Russians listed headaches (p = 0,004).

Among 97% of the respondents, action was taken to treat the common cold and flu – 69% used self-treatment and 28% consulted a general practitioner. In the case of self-treatment, single use of home made remedies and medicinal plants was described as the most common mode of treatment by 56% of the respondents, followed by the combined use of herbal products and OTC medicines.

Statistically significant differences were identified regarding education and treatment activities. Respondents with higher education used more self-treatment with medicinal plants than respondents with different education levels (p = 0.044).

Knowledge about medicinal plants was mostly received from family and friends or literature (49%). Only a few respondents (2%) used general practitioners or pharmacists as an information source about medicinal plants for treatment of the common cold and flu.

### Use of medicinal plants

**Figure 1:**
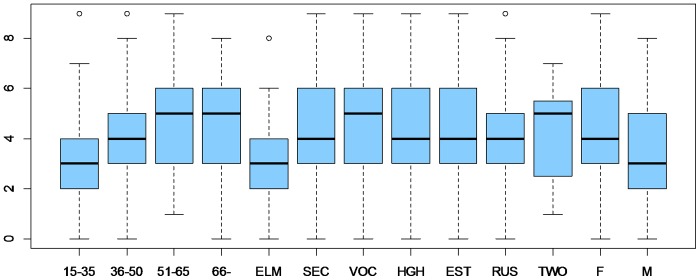
Mean number of used medicinal plants with reference to different demographic characteristics. Abbreviations: age groups: 15–35, 36–50, 51–65, 65+; education: ELM- elementary, SEC – secondary, VOC – vocational, HGH – higher; languages spoken at home: EST – Estonian, RUS – Russian, TWO – both languages; gender: F – female, M – male.

The mean of the total number of plants that respondents had recalled as ever being used for the treatment of episodes of the common cold and flu was 4.1 plants per respondent (Figure 1). The use of medicinal plants was less frequent among respondents with elementary school education (mean 3.1 medicinal plants, p = 0.044) in comparison with other educational groups, and among male rather than female respondents (mean 3.4 medicinal plants, p = 0.022). However, the use of medicinal plants increased with age (p = 0.01).

Respondents have used a combination of Estonian native and non-native species of medicinal plants. Across all the groups of respondents the most used herbs were lemon fruits (73%) and garlic (70%), followed by raspberry (51%), chamomile (48%) and blackcurrant (44%). To some extent ginger (37%), lime flowers (35%) and onion (32%) were used. Only a few people mentioned the use of Iceland moss (11%) and echinacea (9%).

### Age, gender and education

**Figure 2:**
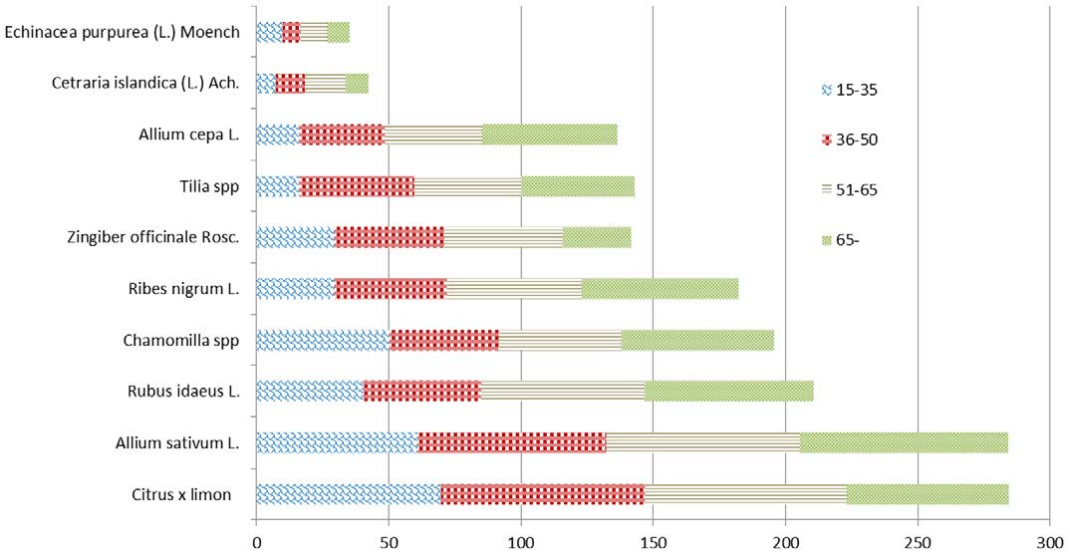
The use of medicinal plants in different age groups. * – p<0.01. ** p<0.05.

In general, consumption of medicinal plants increased with age (p = 0.01). To be specific, the use of garlic (p<0.01), onion, lime flowers, blackcurrant and raspberry increased with age (Figure 2). The use of ginger (p = 0.000), lime flowers (p = <0.013) and blackcurrant (p = 0.005) was more popular among women than men. Respondents with higher education used more lemon (p = 0.005), raspberry and lime flowers (p = 0.011).

### Language spoken

**Figure 3:**
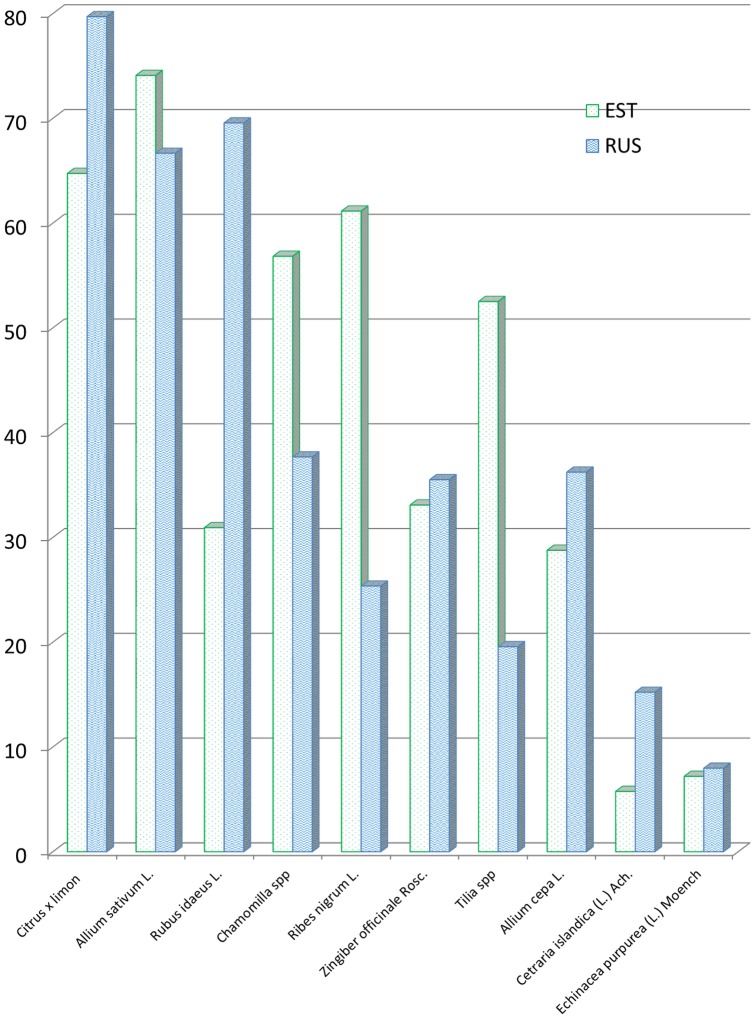
The use of medicinal plants in different language groups. * – p<0.01.

According to the results of the current survey, respondents speaking Estonian were more eager to use lime flowers and chamomile than those speaking Russian. On contrary, the Russian speaking respondents reported more frequent use of raspberry and lemon (Figure 3).

## Discussion

This was the first study in Estonia to describe the use of medicinal plants for the treatment of the common cold and flu by different demographic groups among pharmacy customers. Respondents demonstrated good knowledge in describing the symptoms of the common cold and flu, and a responsible attitude towards the cure of the described minor illnesses. Self-treatment with medicinal plants was widespread, as every respondent used several different species of medicinal plants. Alongside the single use of medicinal plants, home made remedies and OTC medicines were used in combination to relieve the discomfort related to illnesses. The described results support a contemporary approach to healthcare, where complementary medicine and biomedicine have not been contrasted, they further knowledge in biomedicine (e.g. about medicinal products) and even serve as a promoting factor to learn more about medicinal plants and herbal products [18].

The hypothesis set for our survey about differences in the use of medicinal plants by different demographic groups was not always supported by the survey results.

More frequent use of medicinal plants and herbal products at older ages could be connected not only with the insufficient knowledge about medicinal plants of younger generations. Older people suffer more frequently from different illnesses and have the time and motivation to look for different approaches towards relieving their condition. In contrast, younger people look for fast treatment options and combine medicinal plants with OTC medicines.

The findings of our study do not support the results of previous international research where schooling had a negative correlation with the use of traditional medicines [18]. In contrast in our study the respondents with higher education were more frequent users of medicinal plants than the other age groups. The reason for such differences could be connected not with the education itself, but with the environment in which people live and obtain theoretical knowledge and practical skills. Estonia is a small country, not yet strongly influenced by urbanization. Over the last few years there has been a trend towards a more traditional way of living, and respondents with higher education, more knowledgeable in health care issues and in better financial situations, probably practice complementary medicine more frequently.

Nationally based differences in the use of medicinal plants did not always support the information that was previously known. The preference for lemon and garlic contradicts the traditional approach, as in Estonian historical herbal folklore (covering 1888–1994) lemon is never mentioned and garlic is mentioned only few times [5]. Although the medicinal use of garlic was already promoted by some authors in Estonia at the beginning of 20th century, its wider culinary use was established considerably later and its healing properties were finally acknowledged by a popular herbal [19] and its future editions [13].

In our survey the use of garlic depended only on the age of the respondents, contradicting the common myth in Estonia of Russians using this medicinal plant traditionally. Still, the use of lemon depends on the language respondents use and is probably caused by the habit of using lemon in the traditional Russian tea ceremony, which makes lemon more often available in households with a Russian background [20].

The use of lime flowers was unknown in Estonia until the first decades of the 20th century, a historical study of the regional use of plants in the 1930s reports only one use of lime used against colds, among 175 use-reports on the treatment of various diseases [21]. Later, the use of lime became very popular, it became the most used herb against flu and the common cold in the latter half of the 20th century [5],[22].

Despite the modern media emphasizing the use of echinacea for the prevention of flu and the common cold, supported by evidence based data, this herb is the least used by all demographic groups, probably because of its perceived expensiveness: although it is a popular plant in gardens, there is no tradition of making homemade extract, and the respondents used preparations purchased from community pharmacies.

Respondents of the current study used mostly non-professional information sources to obtain knowledge on how to use medicinal plants in the treatment of the common cold and flu. The results are partly supported by earlier research, where previous experience was considered important in decision making about which treatment option to select [15]. However, in Estonia, with its long tradition of the sale of medicinal plants and herbal products at community pharmacies, customers were more likely to employ pharmacists as a professional source of information.

### Survey limitations

The survey was undertaken in only two regions of Estonia, and although an appropriate sample of pharmacy customers was used, the presented results are not generalizable across the whole country or for different demographic groups. The low number of male respondents who participated in the survey could be connected with the lesser frequency of their visits to community pharmacies. In the future, the survey setting could be changed.

## Conclusion

Regardless of some statistically significant differences in preferred species among different age, education, sex and language groups, the general attitude towards medicinal plants for self-treatment of the common cold and flu in Estonia was very favourable. Estonia serves as an example of a country with long traditions of the use of medicinal plants in the treatment of minor illnesses. Due to its geographical location, the traditions of different nations are mixed. In the future, the empirical use of complementary medicine could be more frequently combined with evidence based knowledge about the efficacy of medicinal plants. Future research should evaluate the effectiveness of the most used plants. Moreover, it is important to assess the factors affecting the selection of specific plants, for better understanding of the self-treatment behaviour of the population.
